# Utilising co‐design to develop a lived experience informed personal safety tool within a mental health community rehabilitation setting

**DOI:** 10.1111/1440-1630.12988

**Published:** 2024-08-12

**Authors:** Anna Francis, Amily Le, Karen Adams‐Leask, Nicholas Procter

**Affiliations:** ^1^ SA Health Central Adelaide Local Health Network, Mental Health Clinical Program Adelaide South Australia Australia; ^2^ Clinical and Health Sciences University of South Australia Adelaide South Australia Australia

**Keywords:** co‐design, consumer, lived experience, mental health, occupational therapy, personal safety tool

## Abstract

**Introduction:**

Mental health personal safety tools aim to promote a recovery focus and empower an individualised approach to consumer care. These clinical tools are predominantly utilised in acute mental health settings with a person during or straight after a crisis. There is currently a gap in the literature regarding the preparation of personal safety tools in non‐acute mental health settings. This descriptive article discusses the learnings and outcomes from a co‐designed project that aimed to develop a personal safety tool suitable for a community mental health rehabilitation setting.

**Methods:**

Seven people with lived experience engaging within a mental health community‐based rehabilitation service were recruited through convenience sampling to participate in the co‐design project. A focus group approach was utilised during four group meetings to develop a personal safety tool template. Experiences and ideas about safety planning were transcribed during meetings and thematic analysis extracted key themes. Five steps underpinned the co‐design process that included identifying the need, establishing the co‐design group, planning, design and development, and review and closure.

**Consumer and community involvement:**

Consumer involvement commenced at step two of the co‐design process. The completed personal safety tool was designed with consumer input and review.

**Findings:**

The personal safety tool consisted of nine intervention components. Four key themes emerged from focus group meetings that informed the content of the tool: (i) ensuring the personal safety tool is individualised and meaningful, (ii) promoting exploration of personal strengths and interests, (iii) enabling opportunities to learn self‐management skills, and (iv) treating the personal safety tool as a dynamic and adaptable tool.

**Conclusion:**

Findings suggest that a personal safety tool targeted to a mental health community‐based rehabilitation setting should have an individualised and preventative focus to mental health care. Embedding co‐design principles can support opportunities for meaningful consumer engagement and establishing consumer and clinician partnerships.

**Key Points for Occupational Therapy**

- Personal safety tools within a community mental health rehabilitation setting should promote an individualised and strengths‐based approach.
- Engagement with people with lived experience within occupational therapy quality improvement projects can be enhanced through power sharing and participatory design.
- Quality improvement co‐design projects are enhanced by the integration of rehabilitation and recovery principles.

## 1 INTRODUCTION

A mental health crisis is a state where an individual can feel highly emotionally dysregulated, overwhelmed, with the potential to also experience suicidal ideation (Roennfeldt et al., 2021). Crisis situations can be highly traumatising for individuals and can impact their social, emotional, psychological, and physical functioning (Cassivi et al., 2023). People experiencing a mental health crisis often seek care and treatment from public mental health services (Marynowski‐Traczyk et al., 2013; Morphet et al., 2012).

Interventions to enhance self‐management during times of crises have been a focus of mental health services. These interventions aim to equip people to gain greater control over their mental health symptoms to reduce the incidence and intensity of these experiences. Interventions commonly include learning to anticipate and respond to signs of a crisis and developing skills to manage symptoms and stressors. This can reduce the risk of relapse and repeated acute care admissions following crises (Johnson et al., 2018). One example of an evidence‐based self‐management and crisis prevention strategy is the use of a personal safety tool (PST). Implementing PSTs promote an individualised and recovery focus as well as nurturing valuable and practical therapeutic engagement between professionals and consumers (Chalmers et al., 2012; Moscardini et al., 2020). Studies investigating consumer perspectives have found both safety and joint crisis planning to be beneficial. Consumers have suggested that this is because both approaches focus on interpersonal relationships, communication, and opportunities for developing self‐management skills (Deering et al., 2019; Lequin et al., 2021). Safety planning as an intervention aligns with the values of occupational therapy through its emphasis on working in partnership and person‐centred care. It additionally promotes reduced distress and enhances coping skills, which is likely to mean less interrupted occupational participation and/or more rapid reconnection to preferred meaningful occupations (Marshall et al., 2023).

Well‐known examples of evidence‐based approaches to safety planning include the Safety Planning Intervention by Stanley and Brown (2012) and the development of joint crisis plans (Lequin et al., 2021; Williams et al., 2014). These have predominantly been developed and utilised with a person during or immediately after a mental health crisis (Cassivi et al., 2023; Moscardini et al., 2020). However, there is a lack of information regarding PSTs targeted to a community rehabilitation setting. PSTs have the potential to assist consumers in this setting where there is a unique opportunity to focus on personal strengths, relapse prevention, and skill development.

Partnering with consumers to share their perspectives and expertise is accepted as best practice in service delivery, local quality improvement to policy development (Australian Commission on Safety and Quality in Health Care, 2023; Department of Health, 2011). Occupational therapy and allied health practice have embraced mental health consumer‐led and informed quality improvement and practice initiatives (Aplin & Liddle, 2022; Cox et al., 2020; Whiteford, 2019). Although acceptance and advancement of progressive, participatory consumer engagement frameworks in occupational therapy have improved (Scanlan et al., 2020), the ‘how to’ and ‘know how’ is less well‐developed.

Co‐design is one such participatory approach that can be utilised in mental health service development and design activities. It involves designing, planning, and evaluating actions and outcomes in which consumers, carers, and health professionals work as equal participants (Tindall et al., 2021). The approach values the voices of all who are involved, helps balance power dynamics, and involves the presumption that the product (programs, services, and outcomes) must effectively respond to consumer and carer experiences and interests (Loughhead et al., 2023; Roper et al., 2018). Therefore, utilising a co‐design approach when partnering with people with lived experience in quality improvement projects can enable feeling safe, being heard, and empowered. This project aimed to meet a gap by developing a mental health PST suitable for a community rehabilitation setting using a co‐design approach. This paper aims to describe (i) the lived experience informed PST that was co‐developed and (ii) provide learnings and outcomes from the co‐design process undertaken.

### 1.1 Project background

This project was undertaken within a mental health community clinical rehabilitation service. Consumers attending the service are generally within a stable phase of their illness, and this provides an opportunity to focus on self‐management and skill development. A clinical intervention to support this is through personal safety planning.

The project idea was initiated by occupational therapists who identified that the available safety tools within the service were acute in focus and language and lacked a preventative and rehabilitative focus. A comprehensive review of the literature and consultation with other mental health services failed to identify an existing evidence‐based PST with a community and rehabilitation focus. The literature indicated that mental health safety tools are largely acute and reactive in their focus, and there is currently not a community‐targeted PST that promotes mental health self‐management and skill development.

Safety tools gathered from the literature, other mental health services, and existing service endorsed tools were compiled (Table 1). Occupational therapists proposed to invite people with lived experience to co‐design a PST suitable to the community rehabilitation setting through reviewing and providing feedback on the compiled tools as well as sharing their personal experiences and perspectives in safety planning.

**TABLE 1:** Summary of safety tools reviewed and feedback from people with lived experience.

Existing safety tools reviewed by the co‐design group	Feedback from people with lived experience
Comfort Plan for Wellness and Recovery (Department for Health and Ageing, 2017.)	Hospital focussed in language Very brief prompts for triggers, early warning signs, and coping strategies. It would be helpful to have more options and breakdown the senses Remove history and trauma experiences
Personal De‐escalation Plan (Boston Medical Center, n.d.)	Hospital focussed in language To not include ‘problem behaviours’ Inclusion of strengths and skills is helpful
Safety Planning Intervention (Stanley & Brown, 2012)	Like the wording and language for the headings Helpful to include prompts under the headings Helpful to have option to include more examples
Personal Prevention Plan (Department for Health and Ageing, n.d.)	Hospital focussed in language Prefer having option to include coping strategies at the beginning of the plan
Personal Safety Tool (Cooley Dickinson Hospital, 2006)	Hospital focussed in language Like the title ‘Personal Safety Tool’. Highlights personal/individual focus of the tool To keep triggers, warning signs, and strategies Do not include questionnaire approach
Self‐Regulation and Crisis Intervention Plan Curriculum for Self‐Regulation (Moore, 2015)	Helpful to keep sensory strategies and a variety of options It would be beneficial to break this down into the senses Useful to have the option to include what is calming as well as what you may dislike
The Adolescent Safety Zone Tool (The Massachusetts Department of Mental Health, 2006)	Hospital focussed in language To remove trauma history and restraint prompts Useful to include prompts on safety and what that means and how to feel in control

## 2 METHODS

This is a descriptive article of experiences and learnings of a quality improvement project. A convenience sample of people with lived experience at the community‐based clinical rehabilitation service was recruited on a voluntary basis.

Ethics approval was gained from the Australian and New Zealand Clinical Trials Registry Registration number: ACTRN 12617000901303 and the Human Research Ethics Committee (HREC/17/TQEH/132).

### 2.1 Project setting

The nature of the community rehabilitation service is to support a person to maximise their strengths, skills, and self‐direction. The service is voluntary and aims to provide input to assist the person learn to conduct their own life according to their hopes and aspirations. The staffing profile includes occupational therapists, peer specialist, and an allied health assistant. Eligibility for the service includes being an adult (18–65), residing within the relevant metropolitan catchment area, and having an established mental health diagnosis. Diagnoses are typically severe, complex, and enduring in nature, for example, schizophrenia, bipolar affective disorder, and personality disorders. The service supports consumers from Aboriginal and Torres Strait Islanderand multicultural backgrounds. To access the rehabilitation service consumers must be connected to a public mental health community team. The service promotes lived experience involvement in all service development activities to ensure there is a consumer voice and perspective in how the service and activities are shaped and delivered.

### 2.2 Study design and data collection

A series of four face‐to‐face group meetings were held using a focus group approach (Luke & Goodrich, 2019) to collect feedback and ideas. People with lived experience requested all discussion points from meetings, including brainstorming on butchers paper, be documented in handwritten notes and presented back at the end of each meeting to form a consensus and ensure joint understanding. People with lived experience requested occupational therapists evaluate collected data from group discussions and agreed points to draw out the key themes. As such, a qualitative descriptive study design was adopted using reflexive thematic analysis (Braun & Clarke, 2019).

### 2.3 Data analysis

The principles and processes of thematic analysis (Braun & Clarke, 2019) were drawn upon in this project to ensure that it met the preferences of people with lived experience; however, a rigorous approach was not adopted as this was a small‐scale quality improvement project. Data analysis included three guiding questions to support partnership: (1) what are the perspectives and feedback from people with lived experience on existing safety tools? (2), what are the personal experiences and perspectives of people with lived experience in safety planning? (3), what are the key themes from focus group discussions?. As agreed by the co‐design group occupational therapists transcribed data at step three (planning) and step four (design and development) of the co‐design process during group meetings. The transcribed data and discussion points were read to people with lived experience to ensure the accuracy and credibility of the transcription and data interpretations. All members of the co‐design group were provided copies of transcribed data following each meeting for personal reference and to form a consensus that they agreed with the written content.

### 2.4 Process of co‐design with people with lived experience

**FIGURE 1:**
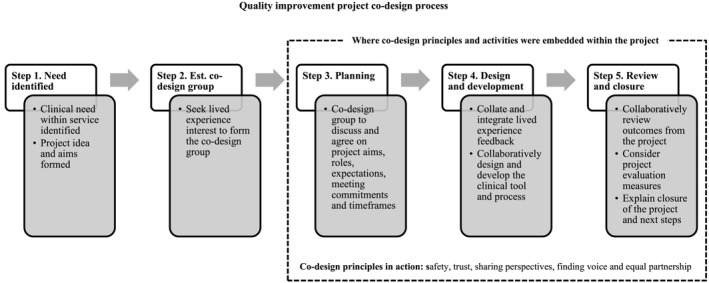
The co‐design process undertaken in this quality improvement project. Steps 3, 4, and 5 demonstrate where co‐design activities and principles were embedded in the project.

The process of co‐design undertaken in this project involved five steps (Figure 1). Co‐design principles and activities that enabled a partnership between people with lived experience and occupational therapists were embedded within steps (3) planning, (4) design and development and (5) review and closure.

#### 2.4.1 Step 1. Need identified

Occupational therapists identified a clinical need for a PST targeted to the mental health community rehabilitation setting to strengthen consumer care. Following a literature review and consultation with other mental health services, it was determined that there was not a safety tool specific to the community mental health rehabilitation setting that led to the project idea. A truly co‐design process was not undertaken at this step of the project as people with lived experience were not involved in the idea conceptualisation.

#### 2.4.2 Step 2. Co‐design group

The project was a collaboration between people with lived experience (*n* = 7) and occupational therapists (*n* = 2). Occupational therapists sought interest from existing consumers engaging in the service who would be willing to participate.

#### 2.4.3 Step 3. Planning

Co‐design principles such as safety, trust, sharing perspectives, and equal partnership (Moll et al., 2020) became embedded at this point of the project. Occupational therapists met with people with lived experience individually to agree upon the project aims, expectations, timeframes, and meeting commitments. It was agreed that a non‐clinical space at the clinical rehabilitation service would be utilised for meetings to facilitate a safe and sharing environment (Isobel, 2021).

The co‐design group agreed to meet weekly for 4 weeks for one and a half hours each meeting. Butchers paper was placed in the middle of the table to take notes from the brainstorming and discussion generated within the meetings. People with lived experience expressed their preference for occupational therapists to bring examples of service endorsed and/or evidence‐based PSTs they had collected and indicated that they did not have any versions or examples that they wanted to personally contribute. People with lived experience discussed their preference to focus on providing feedback and sharing ideas and requested occupational therapists assist with developing prompting questions and scribing. It was further agreed that the compiling, theming, and analysing of discussion during the meetings would be undertaken by the occupational therapists and presented back to the co‐design group after each meeting to ensure joint understanding and accuracy of information. It was agreed that once co‐design group members reviewed and confirmed that they were satisfied with the information presented, it was then provided in a hard copy format for their records. People with lived experience preferred meetings were not audio recorded and requested written notes be taken from the discussions. An opportunity for one‐to‐one debriefing was offered to people with lived experience following meetings.

#### 2.4.4 Step 4. Design and development

##### Meetings 1 and 2

The co‐design group used the first two meetings to review existing safety tools and people with lived experience shared their personal experiences in safety planning. Existing safety tools were selected by occupational therapists from a range of sources as previously agreed. People with lived experience agreed that none of the reviewed tools solely met the need for consumers engaging with the community rehabilitation service because of the acute focus in content and design. The acute‐focussed safety tools were seen as lacking a preventative, personalised, and skill‐focussed approach, which was viewed as a strength for the community rehabilitation setting.

To commence the process of co‐developing the PST, people with lived experience were prompted to consider and feedback to the group (i) what they found helpful and unhelpful of the PST examples, (ii) what they saw as visually appealing or unappealing, and (iii) how these factors may affect their engagement in the process of safety planning. All safety tools that were reviewed can be seen in Table 1 as well as lived experience feedback.

##### Meeting 3

A third meeting was undertaken to collaboratively develop a PST template. This was drawn from feedback reviewing collated safety tools as well as additional experiences and ideas. During this meeting, the co‐design group agreed on naming the template the ‘Personal Safety Tool’ as this was viewed as person‐centred and explained the purpose and individualised focus. The co‐design group discussed options for drafting the PST from the brainstormed notes on butchers paper to an electronic version. People with lived experience requested occupational therapists type the draft and provide copies the following day to allow time to review and edit prior to the final meeting.

#### 2.4.5 Step 5. Review and closure

##### Meeting 4

Occupational therapists prepared a draft PST template for review by the co‐design group who met for a fourth meeting to provide final feedback and edits. A further time was set for the co‐design group to meet following project completion to present the findings and recognise and celebrate the time and contributions of people with lived experience.

### 2.5 Positionality statement

Authors one and two are both experienced occupational therapists with clinical expertise working in a community mental health rehabilitation setting. Authors one and two conceptualised the project and worked collaboratively with people with lived experience to co‐design the PST.

Author three is a mental health occupational therapy discipline lead with expertise in acute, community, and rehabilitation mental health settings. Author four is a clinical academic specialising in mental health nursing and suicide prevention research and education.

Authors three and four have provided supervision and assistance with the interpretation of study findings to authors one and two. All authors have contributed to the manuscript's design and explanation of content.

## 3 FINDINGS

### 3.1 Demographics of people with lived experience

People with lived experience included four males and three females. Their ages ranged from 18 to 58 years with varying mental health diagnoses (Table 2).

**TABLE 2:** Demographics of people with lived experience.

**Age** (years)	18‐58
Median	28
**Gender**	
Male (*n*)	4
Female (*n*)	3
**Primary diagnosis**	
Anxiety (*n*)	1
Depression (*n*)	1
Schizophrenia (*n*)	2
Borderline Personality Disorder (*n*)	2
Schizoaffective Disorder (*n*)	1
**Ethnicity**	
Australian	6
Polish	1

### 3.2 Review and feedback of existing safety tools

Overall, people with lived experience found the safety tool examples to be more focussed to the acute setting with limited rehabilitation references or language. There was a preference to keep language future focussed and remove prompts relating to trauma history. There was also a preference to keep triggers and early warning signs with prompts, as well as keeping sensory strategies with a breakdown of the senses. It was also outlined that existing safety tools did not include options for personal images or ways to personalise the content. There was a preference to have greater flexibility in designing the appearance of the PST, for example, consideration to font, size, and colour.

#### 3.2.1 Personal experiences of safety planning

Previous experiences in safety planning for all seven people with lived experience were limited to inpatient settings. Although they found these experiences helpful, the safety tools felt prescriptive in layout and were not individualised. Most did not recall the specific content or having an opportunity to review their safety tool following the inpatient admission.

#### 3.2.2 Themes informing the content of the PST

Overall, there were four themes that emerged: Ensure the PST is individualised and meaningful; promote exploration of personal strengths and interests; enable opportunities to learn self‐management skills and treat the PST as a dynamic and adaptable tool. Themes were used to inform the content of the co‐designed PST.

##### Theme 1: Ensure the PST is individualised and meaningful

People with lived experience reinforced their preference for the PST to be individualised in content, including options to include personal photos and images and to have choice in the font, colour, structure, and design of their PST. Individualising the PST also meant that consumers have the option to change the size and shape and can transfer copies to other accessible and portable means such as phones or wallet. It was reiterated that personalising the content would make the PST more meaningful and therefore increase motivation to use it.

It will be helpful for the personal safety tool to be available in different sizes and shapes. I would like to keep a picture on my phone or stick the visual prompts in my wallet. (Lived experience participant 1)

I like the idea of including personal pictures, images and colours which I like. This will make the personal safety tool more meaningful and personal for me. (Lived experience participant 3)

##### Theme 2: Promote exploration of personal strengths and interests

Including personal strengths and interests was seen as important. In times of distress, it was discussed that having visual reminders of what and who is important as well as activities of interests and value would be helpful and promote hope. It was suggested that the process of identifying personal strengths in the design of the PST could be therapeutic and increase consumers' self‐confidence.

My personal safety tool needs to be personal to me and have pictures and reminders to look at when I am feeling upset or stressed. (Lived experience participant 4)

It's really helpful for me to be reminded on my personal safety tool of what and who are important in my life rather than what's going on in my mind. (Lived experience participant 7)

##### Theme 3: Enable opportunities to learn self‐management skills

The focus on learning to self‐manage distressing emotions was seen as important. There was a preference to include triggers and early warning signs to help consumers better understand themselves as well as including personalised sensory strategies to manage feelings of distress and feel in control. It was seen as important to include friends, family, or formal supports as a reminder to call for support if needed.

Learning about your strengths and sensory strategies would be very helpful to focus on. It can help you take charge and feel more in control when experiencing a trigger and not going to the emergency department as the first point of call. (Lived experience participant 7)

It's very helpful to have supports to call and people to contact if I need help. This is a good reminder which you can sometimes forget in the moment. (Lived experience participant 5)

##### Theme 4: Treat the PST as a dynamic and adaptable tool

It was important to people with lived experience that the PST is treated as a dynamic and evolving tool that can be updated as individual circumstances and preferences change. This included changing the order in content and structure from the typical safety tool format, for example, commencing with strategies, strengths, and interests. People with lived experience reiterated the importance of providing copies of their PST to nominated supports; however, this could be in a different version and format.

The personal safety tool should change and be updated as things in life change. It needs to remain personal and relevant. (Lived experience participant 2)

We should be able to change the order and appearance on the personal safety tool. I would find it more helpful to look at my strategies, strengths and what's important to me first before looking at early warning signs and triggers. (Lived experience participant 1)

### 3.3 The completed personal safety tool template

**FIGURE 2:**
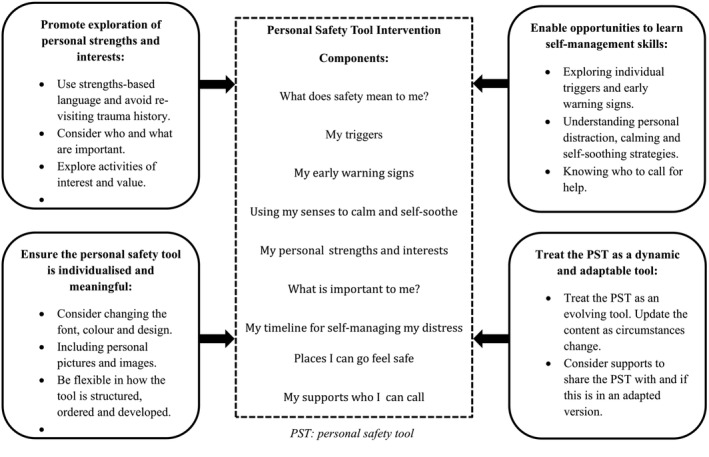
The lived experience informed personal safety tool with key themes that informed the content of the tool.

The completed PST template consisted of nine intervention components that people of lived experience believed held the most value and importance. See Figure 2 for the lived experience informed PST. A full copy can be provided upon request.

### 3.4 Effects of the co‐design process

The co‐design process enabled meaningful engagement, innovation, and initiative. This was evidenced through people with lived experience attending focus group meetings with prepared drawings and self‐reflections. An example of this was a proposed drawing of a visual timeline to support self‐management of distressing symptoms. Another example included the PST being considered in difference shapes and sizes to support the tools' adaptability for a consumer's individual needs.

## 4 DISCUSSION

The purpose of this project was to develop a PST suitable for a mental health community rehabilitation setting utilising a co‐design approach. The co‐developed PST resulted in nine intervention components with a focus on personal strengths, interests, and self‐management strategies. Findings indicated that a PST targeted to a mental health community rehabilitation setting requires a preventative and individualised focus in mental health and should be dynamic in nature, making it unique among existing safety tools. These primary themes as well as the co‐design process undertaken are drawn upon within the following discussion.

### 4.1 A comparison between the co‐designed PST and other safety tools

The intervention components within the co‐designed PST presented with both similarities and unique differences to other published and commonly used safety tools. A systematic review by Marshall et al. (2023) identified similarities across a wide range of existing safety tools that corresponded to the content in the co‐designed PST. These include identifying triggers, warning signs, and supports. The PST content was additionally similar to existing safety tools through its focus on collaborative development and exploring personal and individualised interventions that can be adapted to a person's capacity and resources (Cassivi et al., 2023; Perkins & Repper, 2016).

A key difference to existing safety tools was the focus on personal strengths, values, and the use of preferred colours, fonts, and meaningful images. People with lived experience reinforced the importance of including personal strengths within the community rehabilitation setting to remain strengths‐based and recovery‐focussed in language rather than acute and clinically driven in language. A scoping review by Cassivi et al. (2023, p.1264) found that after reviewing 78 crisis plans, only three included a ‘service users strengths’ section, demonstrating that this is not a commonly included intervention component. This shift of language and focus was considered more relevant and meaningful for the community rehabilitation setting where people are generally in a stable phase of their illness and learning about personal strengths, values, developing self‐management skills, and coping strategies outside of an acute setting. Additionally, the use of existing standardised templates was rejected as they were seen as not allowing personal expression and meaning.

Another difference to the co‐designed PST was its focus to the mental health community rehabilitation setting and being developed with consumers while in the stable phase of their illness to support a preventative approach to relapse. This focus was unique and different from commonly used existing safety tools and approaches, such as safety planning interventions (Stanley & Brown, 2012; Zonana et al., 2018) or crisis planning (Cassivi., 2023), which are intended to be developed with consumers either during or following a mental health crisis.

### 4.2 How co‐design process supported the development of the PST

The co‐design process demonstrated how local quality improvement projects can include people with lived experience to co‐design clinical tools to ensure they reflect consumer needs and preferences. Using co‐design provided a process for people with lived experience to question and challenge existing safety tool intervention components and consider what should be included within the co‐designed PST. It was important to people with lived experience that the PST focussed on individual's strengths, values, and personal resources such as self‐management skills and support networks. Another preference was that the PST move away from reactive conversations about risk management toward preventative and future‐focussed language. These findings align with Perkins and Repper (2016) who proposed what a recovery‐focussed approach to risk versus safety can look like in practice. The study complements the preferences of people with lived experience in identifying that recovery‐focussed safety plans should be centralised around helping individuals lead the lives they wish and build on personal resources to feel greater control.

Another key outcome from the project were the examples of initiative and meaningful engagement in the co‐design process from people with lived experience. The importance of cultivating meaningful consumer roles within service improvement activities is emphasised by Bombard et al. (2018) who suggested that this is a leading factor in what helps maintain consumer engagement within health‐care co‐design activities.

Examples illustrating initiative and meaningful engagement included

- Attending meetings with prepared drawings and reflections.
- Presenting the idea of drawing a timeline that outlines individualised steps and strategies in self‐managing symptoms of mental health distress following a trigger.
- Advocating for the PST to be considered in different shapes and sizes.

### 4.3 The extent to which co‐design principles were implemented

The implementation of co‐design within day‐to‐day clinical activities in government mental health services continues to be an evolving area of development. There remains a need for greater education for staff within local government clinical services to support co‐design in a meaningful and genuine way (Gordon & O'Brien, 2018; Moll et al., 2020).

The literature has suggested that rather than following rigid steps within co‐design, it is more beneficial to follow and enact core principles in co‐design processes. Core principles can include ‘building trust, finding voice, sharing perspectives and creating a common vision for change’ (Moll et al., 2020, p. 3). Co‐design core principles were implemented from the third ‘planning’ stage in this project (Figure 1). This was demonstrated through people with lived experience becoming equal partners in the planning and design and development of the tool. The project deviated from co‐design principles in steps one (need identified) and two (establishing the co‐design group) that were clinician led. Literature suggests that true co‐design involves people with lived experience becoming equal partners from the point of project idea conceptualisation (Gordon & O'Brien, 2018; Steen et al., 2011). Involving consumers from the beginning of the project was an identified challenge of the project setting where quality improvement projects are traditionally clinician initiated. Albeit the clinical need was clinician identified, the content of the PST was ultimately directed by people with lived experience.

A future consideration for local quality improvement initiatives is the implementation of Experience‐Based Co‐design (EBCD) (Chrisholm et al., 2018; Green et al., 2019). EBCD is a more recently developed form of participatory action research aimed at improving health‐care service design and delivery. It involves staff and consumers at each stage of the project including, identifying the problem, process design, and project implementation to work towards a solution (Chrisholm et al., 2018). This approach could be adopted to support consumer engagement from project conceptualisation. Overall, this research was undertaken in a government mental health service that was still striving towards more progressive ways to work with people with lived experience.

### 4.4 Strengths and limitations

The literature exploring co‐design processes within mental health clinical services is evolving, and as a result, there was limited research to use as a guiding framework. In addition, there is an evident gap in translational research exploring how co‐design can be adapted within everyday clinical mental health settings. The lack of involvement of people with lived experience at the project conceptualisation was a limitation of this project. Future projects could consider the EBCD approach where people with lived experience are involved in all stages. Another limitation was the lack of formal evaluation exploring consumer experiences of participating in the co‐design process. Moll et al. (2020) and Esmail et al. (2015) highlight the importance of evaluation within the co‐design process to understand consumer experience and whether the process was meaningful. For example, Arblaster et al. (2023) and Pallesen et al., (2019) used semi‐structured phone interviews following their co‐design studies to evaluate consumer experiences. Despite the lack of formal evaluation within this project, there were opportunities for debriefing at the end of each meeting. Strengths included being a locally led quality improvement project, which means there was relevance to the people of lived experience involved. Co‐design principles were embedded from step three of the project process, which enabled meaningful engagement and strengthened the lived experience and clinician partnership. The co‐development of a PST specifically targeted to the community rehabilitation mental health setting was additionally seen as a strength as this can now be used as a starting point for further development and research.

### 4.5 Future development and research

There are currently plans to undertake a research pilot study to trial the PST and explore its feasibility within the community rehabilitation mental health setting.

## 5 CONCLUSION

The learnings from this quality improvement project contribute to the literature by presenting an innovative and consumer‐centred approach in the way of safety planning within a mental health community rehabilitation setting. The findings from this project additionally demonstrate how the principles of co‐design were utilised in the development of a PST that enabled a partnership between occupational therapists and people with lived experience. Further research exploring the translation of mental health service co‐design processes into clinical practice would be beneficial in promoting growth in this important and evolving area.

## AUTHOR CONTRIBUTIONS

Anna Francis and Amily Le conceptualised the project and worked collaboratively with people with lived experience to co‐design the PST. Karen Adams‐Leask and Nicholas Procter provided supervision and assistance with the interpretation of findings. All authors have contributed to the manuscript's design and explanation of content.

## CONFLICT OF INTEREST STATEMENT

The authors have no conflict of interest to declare.

## Data Availability

The data that support the findings of this study are available from the corresponding author upon reasonable request.
